# Commentary: Impact of systemic immune-inflammation index and systemic inflammation response index on all-cause and cause-specific mortality: a community-based cohort study

**DOI:** 10.3389/fmed.2026.1864968

**Published:** 2026-06-29

**Authors:** Kevin Salvador Rodríguez Quintana

**Affiliations:** Faculty of Human Medicine, Universidad Ricardo Palma, Lima, Peru

**Keywords:** emergency medicine (MeSH database), Latin America, prognosis, sepsis, systemic immune-inflammation index

We read with great interest the study by Ke et al., which identifies the systemic immune-inflammation index (SII) and the systemic inflammation response index (SIRI) as independent risk factors for mortality. We congratulate the authors on this solid contribution that reinforces the utility of complete blood count (CBC)-derived biomarkers in risk stratification (1).

It is noteworthy that both indices demonstrated a significant association with mortality. However, from a pathophysiological standpoint, the biological implications of these biomarkers vary drastically depending on the clinical setting. While Ke et al. analyze chronic, low-grade systemic inflammation as a hallmark of aging within a stable community cohort, acute-care populations present a fundamentally different landscape. In severe acute inflammatory states like sepsis, the SII simultaneously captures hyperinflammation and acute hematologic consumption. In this critical environment, the incorporation of the platelet count into the SII serves as an indirect mirror of microvascular injury and immunothrombosis—where neutrophil extracellular traps (NETs) interact with activated platelets, leading to microvascular occlusion. This acute thrombo-inflammatory cascade and sudden lymphopenia are critical determinants of short-term survival, contrasting with the steady homeostatic decline observed in community settings (2).

Nevertheless, the non-specific nature of the systemic immune-inflammation index constitutes a major clinical limitation that warrants an extremely cautious interpretation. As documented in recent literature, the SII is highly susceptible to confounding by several baseline systemic conditions and subclinical factors outside of acute infectious processes. For instance, the index is significantly altered in chronic metabolic states like cardiovascular-kidney-metabolic syndrome (3) as well as in subclinical infections such as latent tuberculosis (4). Consequently, concomitant hematologic malignancies, active solid tumors, chronic autoimmune disorders, metabolic syndromes, and active corticosteroid or immunosuppressive therapy can heavily skew neutrophil-to-lymphocyte ratios and platelet counts, potentially yielding misleading prognostic classifications in the emergency department.

Consequently, considering the Latin American epidemiological reality—marked by heavy emergency department overcrowding—the SII emerges as a promising, low-cost biomarker of opportunity. However, current evidence gaps in the region demand a more conservative approach. Broader clinical implementation in Latin American emergency and intensive care settings is not yet justifiable without rigorous prospective validation studies and direct, head-to-head performance comparisons against well-established prognostic tools, such as the SOFA or APACHE II scores. Rather than an immediate solution, the SII should be viewed as a potential low-cost adjunct requiring further localized clinical validation before it can safely guide acute therapeutic decisions (5).
